# Suppression of inflammation and tissue damage by a hookworm recombinant protein in experimental colitis

**DOI:** 10.1038/cti.2017.42

**Published:** 2017-10-06

**Authors:** Ivana B Ferreira, Darren A Pickering, Sally Troy, John Croese, Alex Loukas, Severine Navarro

**Affiliations:** 1 Centre for the Biodiversity and Molecular Development of Therapeutics, Australian Institute of Tropical Health Medicine, James Cook University, Cairns, Queensland, Australia; 2 Department of Gastroenterology and Hepatology, The Prince Charles Hospital, Brisbane, Queensland, Australia

## Abstract

Gastrointestinal parasites, hookworms in particular, have evolved to cause minimal harm to their hosts when present in small numbers, allowing them to establish chronic infections for decades. They do so by creating an immunoregulatory environment that promotes their own survival, but paradoxically also benefits the host by protecting against the onset of many inflammatory diseases. To harness the therapeutic value of hookworms without using live parasites, we have examined the protective properties of the recombinant protein anti-inflammatory protein (AIP)-1, secreted in abundance by hookworms within the intestinal mucosa, in experimental colitis. Colitic inflammation assessed by weight loss, colon atrophy, oedema, ulceration and necrosis, as well as abdominal adhesion was significantly suppressed in mice treated with a single intraperitoneal dose of AIP-1 at 1 mg kg^−1^. Local infiltration of inflammatory cells was also significantly reduced, with minimal goblet cell loss and preserved mucosal architecture. Treatment with AIP-1 promoted the production of colon interleukin (IL)-10, transforming growth factor (TGF)-β and thymic stromal lymphopoietin (TSLP), resulting in the suppression of tumour necrosis factor (TNF)-α, IL-13 and IL-17 A cytokines and granulocyte macrophage colony-stimulating factor (GM-CSF), CX motif chemokine (CXCL)-11 and cyclooxygenase synthase (COX)-2 mRNA transcripts. AIP-1 promoted the accumulation of regulatory T cells in the colon likely allowing rapid healing of the colon mucosa. Hookworm recombinant AIP-1 is a novel therapeutic candidate for the treatment of inflammatory bowel diseases that can be explored for the prevention of acute inflammatory relapses, an important cause of colorectal cancer.

Inflammatory bowel diseases (IBD) are a group of chronic autoimmune diseases affecting the digestive track, primarily represented by Crohn’s disease (CD) and ulcerative colitis (UC).^1^ Both types of IBD are caused by an inappropriate immune response in genetically susceptible individuals to intestinal microbial species, however, the site and nature of inflammation differ between the two diseases.^2, 3^ CD can affect the entire intestinal track from the mouth to the anus, whereas UC mainly affects the colon and the rectum. Although the role of innate cells is pivotal in CD, both conditions are T-cell-mediated and characterised by increased levels of interleukin (IL)-6, IL-17, interferon (IFN)-γ and tumour necrosis factor (TNF)-α.^4,5,6^ However, the immune response in UC appears to be more skewed towards a T-helper cell type 2 (Th2) response, with increased levels of IL-4 and IL-13 production in the tissue.^6^ The current treatments for IBD rely on nonspecific immunosuppressive drugs, such as steroids, antibiotics, and immunomodulators targeting the TNF pathway or the gut-homing integrin α4β7.^7, 8, 9, 10, 11^ However, the repetitive cycles of acute inflammation followed by temporary remission in IBD result over time in severe impairment of gut function, motility and tissue remodelling.^12, 13, 14^ Despite encouraging clinical trial end points, TNF-α inhibitors are not effective in all patients and do not prevent relapse.^9, 10, 11^ One of the major consequences of UC progression is the development of colorectal cancer, which is the third most common malignancy in humans.^15, 16^ The rising incidence of IBD parallels the trend of other autoimmune and allergic diseases.^17, 18^ However, for reasons that are still unclear, the rate of childhood-onset IBD has been the highest observed over the past two decades.^19, 20, 21^ In addition to the debilitating symptoms associated with the disease, children affected by early-onset IBD suffer significant malabsorption and nutritional deficiencies resulting in growth failure, skeletal impairment, and significant psychological and developmental delays.^22, 23, 24, 25^ These recent observations underscore the urgent need for novel therapeutic approaches to be developed.

A promising new avenue of research using live helminth therapy has seen encouraging levels of success for the management of autoimmune diseases, such as IBD and Celiac disease.^26, 27, 28^ Indeed, experimental infection with ova of the pig whipworm *Trichuris suis* (TSO) successfully improved both UC and CD Disease Activity Index.^29^ However, because humans are not the natural host and infection resulted in rapid parasite clearance, repeated administrations were required^30^ and recent phase 2 clinical trials in IBD failed to meet clinical end points.^31^ Interestingly, hookworms, such as *Necator americanus and Ancylostoma caninum,* have co-evolved with their mammalian hosts where they establish chronic infections over many years.^28^ The tolerability of iatrogenic *N. americanus* infection has been assessed in patients with autoimmune gastrointestinal diseases. Hookworm infection coupled with escalating oral gluten challenge resulted in remarkably improved gluten tolerance in Celiac patients,^32^ and a non-significant trend towards reduced CD activity scores in a small number of CD patients after hookworm infection.^33^

Nematodes, and hookworms in particular, have been shown to ameliorate chronic inflammatory diseases by promoting regulatory immune circuits, particularly the induction of regulatory T cells (T_regs_) and the modification of the intestinal microbiota.^28, 32, 34, 35, 36, 37^ We, and others, have shown that much of the immunomodulatory prowess of helminths can be attributed to the release of excretory/secretory (ES) products into host tissues.^36, 38, 39, 40^ This complex mix of proteins^41^ and other molecules (unpublished) has been shown to ameliorate colitis in numerous mouse models,^38, 42, 43^ and denaturation of the protein component of ES products ablated the anti-colitic properties.^38^ Proteomic analysis of *A. caninum* excretory/secretory (ES) proteins revealed the relative abundance of two Tissue Inhibitor of Metalloprotease (TIMP)-like proteins, anti-inflammatory protein (AIP)-1 and AIP-2,^41^ neither of which appear to have the protease inhibitory properties that characterise the TIMP family.^44^ We recently showed that AIP-2 induced the expansion of T_regs_ that promoted long-term protection against allergic responses in both mice and humans.^40^ A similar rationale was used earlier to investigate *Ac*-AIP-1 (also referred to as tissue inhibitor of metalloprotease (TIMP)-1) as a potential modulator of dendritic and T-cell function.^45^ However, its efficacy as a therapeutic approach to suppress inflammatory disease was never tested. In this study, haptenating agent 2,4,6-trinitrobenzene sulphonic acid (TNBS) was used to evaluate the therapeutic validity of *Ac*-AIP-1 for treating acute colitis. Despite its limitations, the TNBS-model of colitis is T-cell mediated and skewed towards a Th2 phenotype comparable to human UC.^46, 47, 48^ Recombinant *Ac*-AIP-1 protected against all the hallmark parameters of inducible colitis and promoted a regulatory immune environment in treated mice.

## Results

### Recombinant *Ac-*AIP-1 protects against TNBS-induced intestinal inflammation

**Figure 1:**
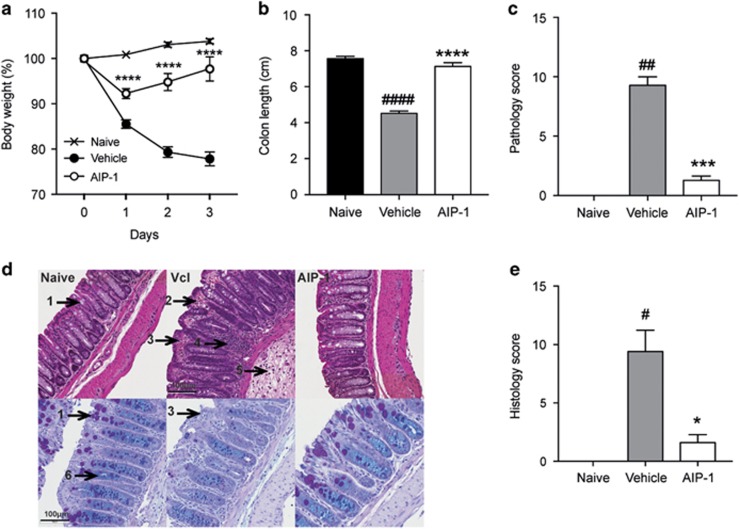
Protective effects of AIP-1 against weight loss and colon damage in TNBS-induced colitis. (**a–e**) Mice received a single intraperitoneal injection (i.p) of 20 μg AIP-1 in PBS or vehicle, followed 5 h later by an enema with 2.5 mg of TNBS in 50% ethanol. (**a**) Body weight was recorded daily for the indicated groups. Data show means±s.e.m. of a representative experiment out of five with *n*=5. Two-way ANOVA with Tukey’s comparisons test used to compare vehicle vs AIP-1 over time. (**b**, **c**) Colons were removed and measured; adhesion, oedema, mucosal wall thickening and ulceration were scored on a scale of 0–3, with three indicating highest degree of damage. (**d**, **e**) Colons were opened longitudinally, washed in PBS, and a 1 cm section from the distal colon was fixed in 4% paraformaldehyde. Data show histological micrographs of hematoxylin and eosin-stained (H&E; top images) or periodic-acid Schiff (PAS; bottom images) (× 200) obtained from a representative mouse from each group. Histological score was performed by assessing epithelial changes (presence of goblet cells (1), hyperplasia (2), erosion (3)), cell infiltrate (4, 5), and mucosal architecture (6). Data show means±s.e.m. of a representative experiment with *n*=5. Mann–Whitney *U-*test performed comparing naïve vs vehicle groups (#) or vehicle vs AIP-1 groups (*); *P<0.05; **P<0.01; ***P<0.001; ****P<0.0001.

The immunoregulatory properties of *Ac*-AIP-1 have previously been explored using an *in vitro* T-cell suppression assay and protein-pulsed bone-marrow-derived dendritic cells.^45^ Suppression of inflammation using the related hookworm protein, AIP-2, was shown to be optimal at 1 mg kg^−1^ in a mouse model of asthma^40^ and colitis (unpublished). Therefore, the same dose for administering AIP-1 herein was used. To determine the therapeutic potential of AIP-1 in a model of acute inflammation, mice were treated with 1 mg kg^−1^ of AIP-1 or vehicle via the intraperitoneal (i.p) route. Five hours later, mice were administered 2.5 mg trinitrobenzoylsulfonic acid (TNBS) in 50% ethanol via intrarectal injection, resulting in a 15 to 20% weight loss in the vehicle group over the course of the study (Figure 1a). Interestingly, mice treated with AIP-1 displayed <10% weight loss on the first day post-TNBS injection, and recovered most of their initial weight by day 3 (Figure 1a). Cellular enumeration of peritoneal lavages showed that i.p treatment with AIP-1 did not induce eosinophil infiltration at the site of injection (data not shown). In comparison to the naive control mice, colon lengths were significantly decreased in the vehicle group (*P*<0.0001), while AIP-1-treated mice remained unaltered by the administration of TNBS (Figure 1b). Macroscopic analysis of the colons revealed a significant reduction of tissue inflammation as seen by minimal adhesion, oedema, wall thickening and ulceration (Figure 1c). Haematoxylin and eosin (H&E) staining of distal colon sections from the vehicle group showed mucosal erosion and epithelial hyperplasia, pronounced cellular infiltration in the lamina propria and intraepithelial compartments, evidence of oedema and ulceration, and loss of healthy goblet cells (Figure 1d, top panels, Figure 1e). However, mice treated with AIP-1 displayed an overall mucosal architecture similar to that of healthy controls (Figures 1d and e). Mucin secretion and goblet cell numbers following periodic acid-Schiff-alcian blue (PAB) staining of the colons further illustrated that AIP-1 treatment promoted the maintenance of mucosal barrier integrity (Figure 1d, bottom panels). In contrast, vehicle-treated mice displayed a significant decrease in mucin production, loss of goblet cells and pronounced mucosal barrier remodelling (Figure 1d, bottom panels). Together, these results show that AIP-1 is highly efficient at suppressing TNBS-induced intestinal inflammation.

### Production of *N*-glycan-deficient *Ac-*AIP-1

**Figure 2:**
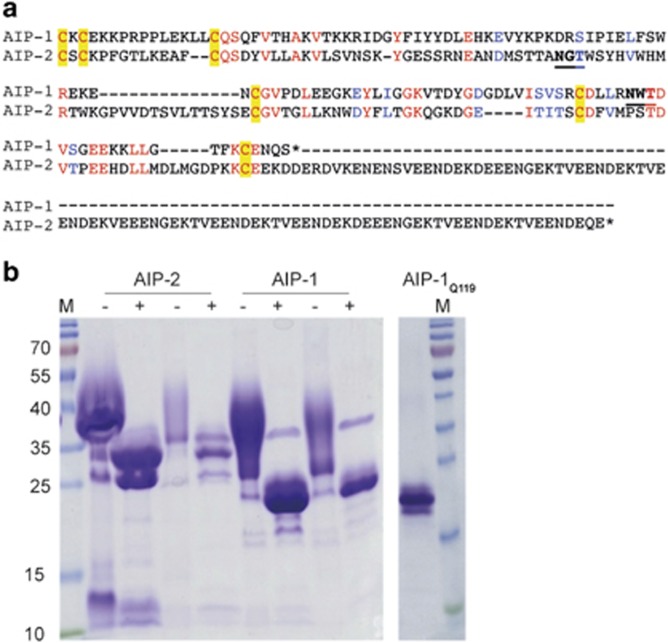
Wild type *Ac*AIP-1 expressed in *Pichia* is glycosylated at Asn-119, and mutation of Asn-119 to Gln ablates *N*-glycosylation of the recombinant protein. (**a**) Primary sequence alignment of the mature (signal peptides removed) sequences of *Ac*AIP-1 and *Ac*AIP-2. Conserved residues are shown in red and similar substitutions are in blue. Disulfide-forming Cysteines are in yellow boxes. *N*-glycosylation sites (where the Asn was substituted for Gln) are underscored and in bold font. Note the long acidic C-terminus of AIP-2 that is absent in AIP-1. (**b**) The image on the left shows a SDS-polyacrylamide gel stained with Coomassie blue showing recombinant purified wild type AIP-1 and AIP-2 treated (+) or not (−) with PNGaseF to remove *N*-glycans. Five and one microgram of each protein was loaded on the gel. The image on the right shows recombinant AIP-1Q119 (1 μg); note its molecular weight is similar to enzymatically deglycosylated wild type AIP-1. Molecular weight markers (M) are shown on the left and right.

Yeast-based expression of recombinant proteins is associated with high *N*-linked glycosylation, which can interfere with the immune system.^46, 49, 50, 51, 52^ In addition, helminth excretory/secretory (ES) products contain glycan moieties that have been shown to skew the immune system, particularly towards the Th2 phenotype.^52^ The native AIP-1 protein sequence contains an asparagine (Asn) at position 119, which appears to be glycosylated by *Pichia* (Figures 2a and b). To eliminate addition of the *N-*glycan we substituted Asn-119 for glutamine (Gln), and termed the mutant recombinant protein AIP-1_Q119_. SDS-PAGE profile of AIP-1_Q119_ showed an absence of smearing compared to wild type recombinant AIP-1, indicative of an absence of *N*-glycosylation (Figure 2c).

### AIP-1-induced protection against intestinal inflammation is independent of yeast-derived glycan modification

**Figure 3:**
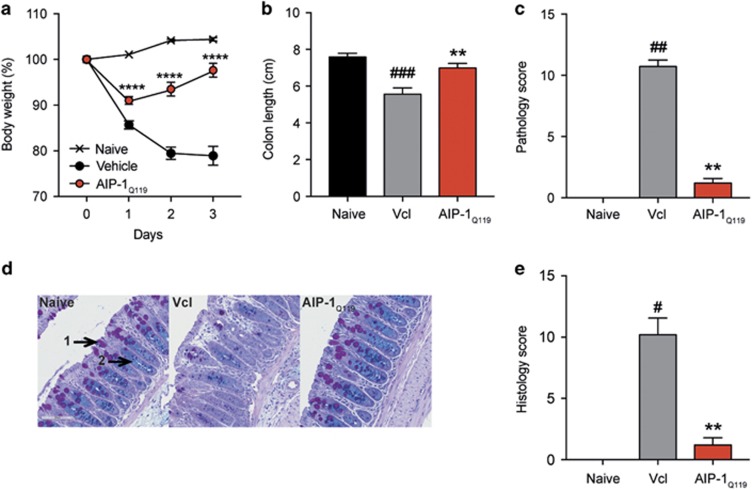
AIP-1_Q119_ retains the protective properties against TNBS-induced inflammation. (**a–e**) Mice received a single intraperitoneal injection (i.p) of 20 μg AIP-1_Q119_ in PBS or vehicle, followed 5 h later by an enema with 2.5 mg of TNBS in 50% ethanol. (**a**) Body weight was recorded daily for the indicated groups. Data show means±s.e.m. of a representative experiment out of five with *n*=5. Two-way ANOVA with Tukey’s comparisons test used to compare vehicle vs AIP-1_Q119_ over time. (**b**,**c**) Colons were removed and measured; adhesion, oedema, mucosal wall thickening and ulceration were scored on a scale of 0–3, with 3 indicating highest degree of damage. (**d**,**e**) Colons were opened longitudinally, washed in PBS, and a 1 cm section from the distal colon was fixed in 4% paraformaldehyde. Data show histological micrographs of periodic-acid Schiff (PAS) (× 200) obtained from a representative mouse from each group. Histological score was performed by assessing epithelial changes (presence of goblet cells (1), hyperplasia (2), erosion), cell infiltrate, and mucosal architecture. Data show means±s.e.m. of a representative experiment out of five, with *n*=5. Mann–Whitney *U-*test performed comparing naïve vs vehicle groups (#) or vehicle vs AIP-1_Q119_ groups (*); *P<0.05; **P<0.01; ***P<0.001; ****P<0.0001.

To assess whether Asn-119 substitution to Gln affected the anti-inflammatory properties of the recombinant wild type protein, mice were treated as described previously with AIP-1_Q119_ (1 mg kg^−1^) or vehicle prior to TNBS injection (Figures 3a–e). The level of protection in AIP-1_Q119_-treated mice against weight loss was comparable to that of AIP-1 (Figure 3a). Colon shortening and pathology were also significantly inhibited with AIP-1_Q119_ when compared to the vehicle control (Figures 3b and c). Finally, mucosal architecture and integrity were maintained upon treatment in comparison to the vehicle group as shown by the significant reduction of histology score in AIP-1_Q119_-treated mice (Figures 2d and e).

### Treatment with AIP-1 or AIP-1_Q119_ suppresses systemic inflammation

**Figure 4:**
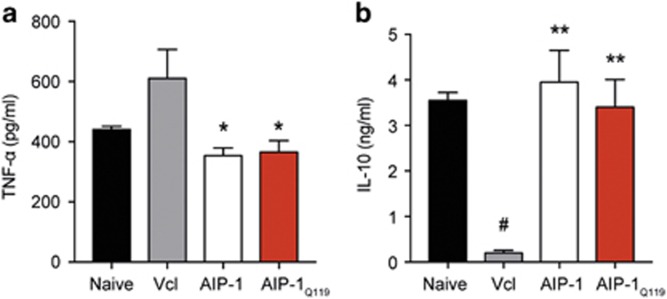
AIP-1 and AIP-1_Q119_ modulate systemic cytokine production in TNBS-treated mice. (**a**, **b**) Culture supernatant concentrations of TNF-α and IL-10 assessed by ELISA from splenocytes stimulated with 1 μg ml^−1^ α-CD3 for 3 days. Data show means±s.e.m. of a representative experiment out of five, with *n*=5. Mann–Whitney *U-*test performed comparing naïve vs vehicle groups (#) or vehicle vs AIP-1 or AIP-1_Q119_ groups (*); *P<0.05; **P<0.01.

*In vitro* T-cell receptor stimulation of splenocytes revealed a significant systemic inhibition of tumour necrosis factor (TNF)-α production in both AIP-1- and AIP-1_Q119_-treated mice, which correlates with the suppression of TNBS-induced pathology observed previously (Figure 4a). Interestingly, both recombinant proteins seemed to restore systemic production of IL-10 to a level comparable to that seen in healthy controls, suggesting the promotion of pro-regulatory responses by AIP-1 (Figure 4b).

### AIP-1_Q119_ promotes colon immune regulation and tissue repair

**Figure 5:**
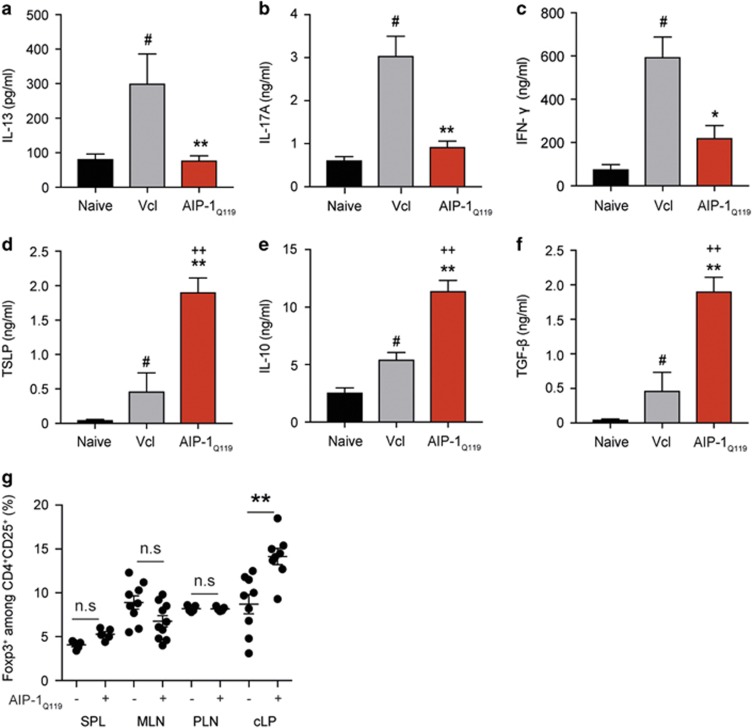
AIP-1_Q119_ induces a pro-regulatory environment in the colon of mice exposed to TNBS. (**a–f**) Concentrations of IFN-γ, IL-10, IL-13, IL-17 A, TGF-β and TSLP assessed by ELISA on colon lysates. Data show means±s.e.m. of a representative experiment out of five, with *n*=5. Mann–Whitney *U*-test performed comparing naïve vs vehicle groups (#), naive vs AIP-1_Q119_ groups (+), or vehicle vs AIP-1_Q119_ groups (*); *P<0.05; **P<0.01. (**g**) Naive mice were treated with daily intraperitoneal injections of 20 μg AIP-1_Q119_ for 4 days, indicated tissues were collected 24 h after the last injection, and prepared for flow cytometry analysis. SPL, Spleen; MLN, mesenteric lymph nodes; PLN, peripheral lymph nodes (popliteal, inguinal and brachial); cLP, colon lamina propria. Data show the frequency of Foxp3-expressing cells among CD4^+^CD25^+^ cells for individual mice from a representative experiment with *n*=9–10 out of 2. Mann–Whitney *U*-test performed comparing vehicle vs AIP-1_Q119_ groups (*); NS, non-significant; **P<0.01.

To address the impact of AIP-1_Q119_ treatment on the production of cytokines at the site of inflammation, colons of mice exposed to TNBS were homogenised and analysed by ELISA (Figures 5a–c). In line with our previous observations, AIP-1_Q119_ treatment significantly suppressed inflammatory cytokines IL-13, IL-17 A and IFN-γ (Figures 5a–c). Interestingly, AIP-1_Q119_ administration also significantly increased the levels of thymic stromal lymphopoietin (TSLP) in the colon suggesting mucosal healing (Figure 5d). As seen previously in the spleen, IL-10 production in the colon of AIP-1_Q119_-treated mice was also markedly increased as well as TGF-β, suggesting the promotion of regulatory responses (Figures 5e and f). To reveal the potential involvement of regulatory T cells (T_reg_) in the protection against TNBS-induced colitis by AIP-1_Q119_, colons and peripheral tissues were collected and cells analysed by flow cytometry. While no significant differences were seen in the peripheral tissues, colons of mice treated with AIP-1_Q119_ displayed a significantly increased frequency of CD4^+^CD25^+^Foxp3^+^ cells (Figure 5g).

### AIP-1_Q119_ affects the proinflammatory processes induced by TNBS in the colon

**Figure 6:**
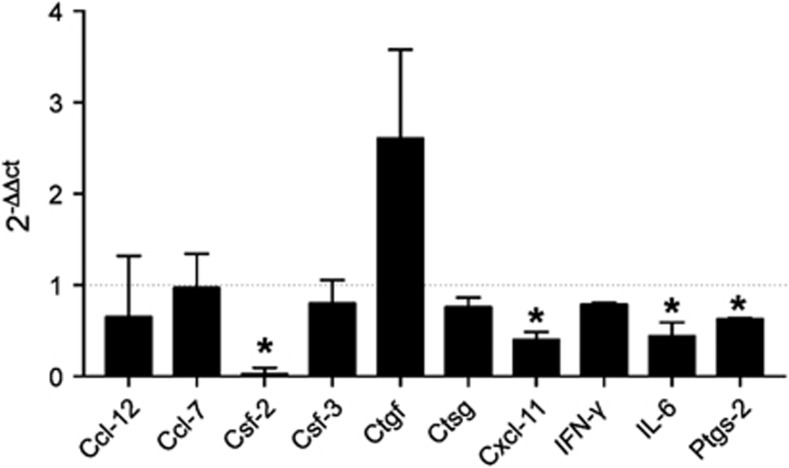
AIP-1_Q119_ modulates colon gene expression profile in TNBS-exposed mice. Total medial colon mRNA transcript levels of 10 inflammatory genes were measured at day 4. Data show the average relative gene expression levels (2^ΔΔCT^) of AIP-1_Q119_-treated mice compared to vehicle, with n=5. A 2^ΔΔCT^ of 1 representing no change in expression level (dotted line). Mann–Whitney *U-*test was used to determine statistical significance compared with the vehicle group. *P<0.05.

To reveal suppression of inflammation by AIP-1_Q119_ in gut tissue, total RNA was extracted from colon sections of mice exposed to TNBS. Differentially expressed genes were identified by comparison of expression levels with vehicle-treated samples that served as baseline (Figure 6). Mice treated with AIP-1_Q119_ displayed a significant decrease in the expression of mRNAs in the colon encoding the proinflammatory mediators granulocyte macrophage colony-stimulating factor (GM-CSF)- 2, cyclooxygenase (COX)-2, IL-6 and CXC-motif chemokine 11. Although not significant, mice treated with AIP-1_Q119_ also displayed increased expression of connective tissue growth factor (CTGF)-encoding mRNA, indicating potential enhanced tissue repair processes. Together, this profile suggests that AIP-1_Q119_ affects the expression of factors responsible for the migration of activated inflammatory cells such as neutrophils and lymphocytes, likely contributing to the reduction of tissue damage and promoting repair.

## Discussion

We^38, 42^ have previously demonstrated that *A. caninum* excretory/secretory (*Ac*ES) products significantly alleviated intestinal pathology in a mouse model of UC. Protease digestion and heat denaturation showed the protective compound(s) of *Ac*ES were of protein moieties. Interestingly, despite *Ac*ES retaining characteristics of inducing Th2 cytokines, pro-regulatory and repair mechanisms were also observed.^38^ Indeed, proinflammatory cytokines IFN-γ, IL-6, IL-17 A and TNF-α were dramatically suppressed, and the recruitment of alternatively activated macrophages and IL-10/IL-4-producing cells were seen in the mucosa. As previously, shown by Mulvenna and colleagues, *Ac*ES is a complex mixture of over 100 proteins, the function of which remain mostly unknown.^41^ Recently, one of the most abundant proteins produced by *A. caninum*, AIP-2, was shown to suppress allergen-induced inflammation both in experimental asthma and PBMCs from confirmed allergy patients.^40^ AIP-2 administration modified mesenteric dendritic cell function by enhancing retinaldehyde dehydrogenase activity, resulting in a significant accumulation of T_regs_ at mucosal sites. Interestingly, *A. caninum* AIP-1 is also an abundant protein found in *Ac*ES; however, its function remains unclear. *Ac-*AIP-1, like *Ac-*AIP-2, contains a signal peptide followed by a TIMP-like netrin domain. *Ac-*AIP-2 has a C-terminal tail that appears to be absent from *Ac*-AIP-1 (Figure 2). Cuellar and colleagues showed that AIP-1 induced a systemic state of T-cell unresponsiveness as seen by *ex vivo* T-cell receptor (TCR) activation of splenocytes with anti-CD3.^45^ The protein was further shown (in a non-diseased state) to modulate major histocompatibility complex (MHC)-II expression on bone-marrow-derived dendritic cells which were capable of inducing IL-10-producing CD4 and CD8 Foxp3^+^ T_regs_.

In this study, we have demonstrated that *Ac-*AIP-1 significantly protected mice against TNBS-induced weight loss, as well as the cardinal features of colitis. Indeed, colon mucosal barrier integrity was maintained despite the chemical assault resulting in the absence of colon shortening, oedema, ulceration and necrosis. A mutation on the glycosylation site of the recombinant protein demonstrated that the protection induced by AIP-1 was not a bystander immunomodulatory effect of glycans added during the protein expression and folding process in *Pichia*.^51, 52^ When administered systemically in TNBS-treated mice, both native and glycosylation mutant AIP-1 significantly suppressed the production of IL-13, IL-17 A, IFN-γ and TNF-α, known to play a central role in IBD.^53, 54^ Although IL-13 is directly implicated with increased colon epithelial permeability and apoptosis, therapeutic intervention using IL-13 blockade, Anrukinzumab, failed to show any improvement of clinical response or remission.^55, 56^ Evidence undeniably supports that colitis is a multifactorial disease, which cannot be suppressed with the neutralisation of a single component, such as IL-13. However, inhibition of signal transducer and activator of transcription (STAT)-6, for which the phosphorylation status is highly elevated in UC patients, seemed to prevent IL-13-induced apoptosis and improved transepithelial resistance.^57^ Because of the complexity of IBD pathology, cytokine neutralisation therapy has shown mixed results suggesting that the development of novel treatments should focus on different targets. Indeed, the use of Infliximab or Adalimumab, both monoclonal antibodies directed against TNF-α, were shown to be highly effective against acute colitis, although efficacy was strongly dependent on the severity of the disease.^58, 59^ In addition, resistance to anti-TNF-α therapy has been observed over time, supporting the notion that single cytokine therapy is insufficient for the treatment of IBD. Indeed, the suppression of proinflammatory mediators without promoting mucosal barrier repair will not prevent relapse and disease progression. Interestingly, despite the short timeline of the TNBS model, AIP-1 not only suppressed key pathogenic cytokines important to colitis, but also significantly prevented colonic mucosal damage.

As seen previously with *Ac*ES treatment in experimental colitis, AIP-1 promoted the production of IL-10, suggesting a potential mechanism of systemic regulation of inflammation.^38^ A similar observation was made upon analysis of the colonic mucosa in which the levels of IL-10 were significantly elevated in comparison to vehicle-treated mice but also in comparison with naïve control. This further supports the notion that IL-10 production seems to be an important suppressive mechanism induced by AIP-1. A genetic-linkage analysis of patients with colitis revealed distinct mutations in the IL-10 gene, demonstrating a central role for this cytokine in the negative feedback necessary to maintain mucosal homeostasis.^60, 61^ In our study, TGF-β was also found to be elevated in the colonic mucosa upon AIP-1 treatment. Not only is TGF-β pivotal for the suppression of gut inflammation and enhancing barrier function, but it also suppresses tumour progression in colon cancer and promotes the induction of functional T_regs_ from naive CD4^+^ T-cell precursors.^62, 63^ Interestingly, treatment with AIP-1 resulted in a significant increase in the frequency of CD4^+^CD25^+^Foxp3^+^ T cells in the colonic lamina propria. Together, this data suggests that AIP-1 modulates the mucosal cytokine environment by enhancing regulatory processes.

Despite its involvement in the development of allergy and key role in the induction of Th2 responses, TSLP has been shown to have protective effects in experimental colitis.^64, 65^ The levels of TSLP in the colon of AIP-1-treated mice were strikingly higher than both naïve and vehicle control mice. Interestingly, TSLP was shown to be important for protective immunity following infection with the gastrointestinal nematode *Trichuris muris* by limiting Th1- and Th17-induced mucosal damage.^65, 66, 67^ In addition, TSLP can induce Foxp3^+^ T_reg_ by influencing plasmacytoid dendritic cell function in both mice and humans.^68, 69^ AIP-1 administration modulated colon expression of a combination of mediators involved in inflammation; expression of mRNAs encoding GM-CSF, CXCL11, IL-6 and PGE-2 were significantly downregulated in AIP-1-treated mice in comparison to vehicle control. Reduced expression of these particular mediators implies that AIP-1 may affect the expansion and migration of activated Th1/Th17 cells induced by TNBS by interfering with PGE-2/IL-6 signalling and GM-CSF/CXCL11 production.^70^ Although not significant, the expression level of connective tissue growth factor (CTGF) trended towards elevated levels in AIP-1-treated mice, further supporting cell turnover, wound healing and tissue repair.^71^ While the direct action of AIP-1 on the epithelium, the production of TSLP and the inhibition of PGE-2 has yet to be fully demonstrated, AIP-1 seems to induce a multifactorial response beneficial for the suppression of inflammation and tissue damage induced by TNBS.

In like fashion to our findings with AIP-2 in a mouse model of asthma,^40^ AIP-1 seems to promote regulatory cells in the mucosa and suppress inflammation.^40^ However, the upregulation of genes involved in mucosal turnover observed herein for AIP-1 was not described for AIP-2. Indeed, AIP-1 seems to have a role in modulating local and systemic production of pro-regulatory cytokines, such as IL-10 and TGF-β, likely allowing tissue repair. Another cytokine important for mucosal repair in IBD is IL-22, which was significantly elevated in the colon of human volunteers experimentally infected with human hookworms.^37^ However, the involvement of IL-22 in AIP-1-induced protection in colitis has yet to be determined. Considering that both AIP-1 and AIP-2 are found abundantly in *Ac*ES, one can postulate that both proteins act concertedly to increase the number of regulatory cells and allow the tissue to rapidly heal from parasite-induced injury. While it would be pertinent to assess the combined therapeutic role of AIP-1 and AIP-2 in colitis, we have shown here that on its own, AIP-1 seems to be a good therapeutic candidate for the treatment of colitis by supressing inflammatory responses, preventing tissue remodelling and promoting gut healing.

## Methods

### Mice

Five-week-old male C57BL/6 were purchased from the Animal Resources Centre (Perth, Western Australia, Australia) and were housed according to Australian code for the care and use of animals for scientific purposes under specific pathogen-free conditions. Mice received food and water *ad libidum*. All procedures were approved by the James Cook University Animal Ethics Committee under projects A1484 and A2012.

### Reagents and protein expression

Recombinant *Ac*-AIP-1 and the glycosylation mutant *Ac-*AIP-1_Q119_ were expressed as secreted proteins in the yeast *Pichia pastoris* using methods described elsewhere.^72^ Mutation of Asn-119 to Gln was achieved using PCR as described elsewhere.^73^ The cDNAs encoding the mature sequences of *Ac*-AIP-1 (amino acids 17-140) and *Ac*-AIP-1_Q119_ were cloned in frame into pPICZαA (Invitrogen, CA, USA) using *Xho*I and *Xba*I restriction sites. The recombinant plasmids were linearized by *Sac*I digestion and transformed into *P. pastoris* strain X-33 by electroporation according to the manufacturer’s instructions (Invitrogen). Transformants were selected on yeast extract-peptone-dextrose plates containing zeocin and assessed for expression of recombinant protein via western blot with monoclonal anti-6 × His antibody. A western blot-positive clone for each protein was grown in a shaker flask, and expression of the recombinant 6 × His tagged *Ac*-AIP-1 and *Ac*-AIP-1_Q119_ were induced with methanol, as per the manufacturer’s instructions (Invitrogen). The recombinant fusion proteins were purified with a nickel affinity column and eluates containing *Ac*-AIP-1 and *Ac*-AIP-1_Q119_ were concentrated using Amicon Ultra Centrifugal concentrators and buffer exchanged into phosphate-buffered saline (PBS) pH 7.4. Lipopolysaccharide contents in *Ac*-AIP-1 and *Ac*-AIP-1_Q119_ were below 5 ng mg^−1^ as determined by the Limulus Amoebocyte Lysate (LAL) assay (Pierce Thermo Fisher Scientific, MA, USA).

### Induction of colitis

Mice were randomly assigned to each group. Recombinant proteins were administered via the intraperitoneal (i.p) route in sterile phosphate-buffered saline at a dose of 1 mg kg^−1^. Five hours later, mice were anaesthetised with xylazine (5 mg kg^−1^, Rompun 2%, Bayer, Germany) and ketamine (50 mg kg^−1^, Ketavest; Pfizer Inc., NY, USA). 2,4,6-Trinitrobenzenesulfonic acid (TNBS; Sigma-Aldrich, MI, USA) was prepared by dissolving 2.5 mg in 50% ethanol. Once unresponsive, mice received an enema with a 125 mg kg^−1^ dose of TNBS using a lubricated 20-G soft catheter (Terumo, Tokyo, Japan) as previously described.^47, 48, 74^ Animals were monitored daily for weight loss and general wellbeing over 4 days. Colitis experiments were repeated five times with a sample size (*n*) of five mice per experimental group.

### Clinical assessment of colitis

To eliminate bias, mice were assessed in a blinded fashion and de-identified at end point. Mice were weighed daily, and their overall appearance (piloerection), activity level and posture were recorded. No animals were excluded from the study. On day 3 following TNBS injection, mice were killed by CO_2_ asphyxiation, and colons were collected for observation, characterisation by flow cytometry, cytokine measurements and RNA extraction. When dissecting, the level of tissue adhesion was scored from 0 to 3, with 0 corresponding to absence of adhesion and 3 corresponding to severe adhesion. Colons were measured, cut longitudinally, washed in saline, and observed under an Olympus SZ61 microscope (Notting Hill, VIC, Australia) (× 0.67–4.5). Scoring of clinical pathology included adhesions (0–3), mucosal oedema (0–3), ulceration (0–3) and bowel wall thickening (0–3), for a maximum total score of 12 as previously described.^38^

### Tissue preparation and cell culture

Mesenteric lymph nodes (MLN), peripheral lymph nodes (brachial, inguinal, and popliteal) (PLN), spleens and colons were processed in RPMI 1640 media containing 2% foetal bovine serum (FBS), 400 U type I collagenase and 1 mg ml^−1^ DNase I (Life Technologies, Thermo Fisher Scientific, MA, USA) using GentleMACS (Miltenyi Biotec, Germany) and incubated for 15 min at 37 °C. Cells were strained through a 70 μm cell strainer (BD Biosciences, NJ, USA). Erythrocytes were lysed with red blood cell lysis buffer (ACK). Colon lamina propria (cLP) were obtained after digestion in RPMI containing 5% FCS, 5 mm EDTA, and 2 mm dithiothreitol (DTT) as described previously.^75^ Briefly, colons were washed in ice-cold PBS, minced and incubated under agitation for 30 min at room temperature. Intestinal epithelial lymphocytes were discarded by filtration and the remainder was further incubated in RPMI containing 5% FBS, 400 U type I collagenase and 1 mg ml^−1^ DNAse I for 30 min at 37 °C. Cells were filtered and stained with anti-mouse CD3, CD4, CD25 and Foxp3 monoclonal antibodies (BD Biosciences, eBiosciences Thermo Fisher Scientific) and analysed on a BD FACSCanto II flow cytometer. T_reg_ enumeration experiments in which naïve mice were treated with a daily i.p injection of AIP-1_Q119_ or vehicle for 5 days were repeated twice with a sample size (*n*) of nine mice (vehicle) and ten mice (AIP-1_Q119_).

### Histology

Distal colons were collected on day 3 post-TNBS injection and fixed overnight in 4% paraformaldehyde, dehydrated with 70% ethanol and embedded in paraffin. Sections were stained with haematoxylin and eosin (H&E) for morphology or periodic acid Schiff for detection of mucopolysaccharide accumulation as described previously.^76^

### Cytokine quantification

Splenocytes were cultured in triplicate in flat-bottom 96-well plates (10^6^ cells per well) either with complete RPMI 1640 medium alone or in medium supplemented with 1 μg ml^−1^ anti-CD3 antibody (BD Biosciences) for 72 h at 37 °C and 5% CO_2_. Colon samples were homogenized in calcium- and magnesium-free Hank’s Balanced Salt Solution and phosphatase and protease inhibitor cocktail (Roche, Basel, Switzerland). IFN-γ, TNF-α, IL-10, TGF-β (latent and active form), TSLP, IL-13 and IL-17 A were quantified by ELISA (BD Biosciences) from splenocyte supernatants and colon homogenates.

### RNA extraction and gene array

A colon section (0.5 cm) was washed in PBS, placed in 1 ml of TRIzol and dissociated using a TissueLyser (Qiagen, Hilden, Germany) for 10 min with the use of metal beads. Total RNA extraction was performed by phenol–chloroform separation according to the manufacturer’s instructions. After treatment of RNA with RQ1 DNase (Promega, WI, USA), first-strand cDNA was produced with random hexamers and SuperScript III reverse transcriptase (Invitrogen). Samples were tested in 1:100 dilution using a custom wound healing RT2 profiler PCR array and SYBR green (Qiagen). A Rotor-Gene 6000 (Qiagen) was used for real-time thermal cycling. Melting curve analysis was used to confirm that single products had been amplified. All genes were normalised for levels of transcription relative to the housekeeping genes beta-glucuronidase (*Gusb*), Hypoxanthine guanine phosphoribosyl transferase (*Hprt*), Glyceraldehyde-3-phosphate dehydrogenase (*Gapdh*) and beta-actin *Actb*.

### Statistical analyses

All data were analysed with GraphPad Prism (version 7; San Diego, CA, USA). Sample size (*n*=5) was determined by using a power of 80%, one-sided test, representing the probability of finding significant differences between vehicle and AIP-1 or AIP-1_Q119_-treated groups, with an acceptable Type 1 error of 0.05 and an expected effect size of 1.8. Data are expressed as the mean±s.e.m. Body weight values were analysed using two-way analysis of variance (ANOVA) followed by the Tukey’s *post-hoc* test. Comparisons for all pairs were performed by unpaired two-tailed Mann–Whitney *U-*test. Significance levels were set at a *P* value of 0.05.
